# 

**Published:** 1871-08

**Authors:** 


Vol. XII. AUGUST, 1871. No. 8.

### CHICAGO Medical Examiner,

N. S. DAVIS, M.D., Editor, and

F. H. Davis, M.D., Assist.-Editor.

TERMS, $3.00 PER ANNUM, IN ADVANCE.

CHICAGO:
ROBERT FERGUS' SONS, PRINTERS,
12 CLARK AND 242—248 ILLINOIS STREET.
1871.

